# The cotton *GhWIN2* gene activates the cuticle biosynthesis pathway and influences the salicylic and jasmonic acid biosynthesis pathways

**DOI:** 10.1186/s12870-019-1888-6

**Published:** 2019-08-28

**Authors:** Xiancai Li, Nana Liu, Yun Sun, Ping Wang, Xiaoyang Ge, Yakun Pei, Di Liu, Xiaowen Ma, Fuguang Li, Yuxia Hou

**Affiliations:** 1 College of Science, China Agricultural University, No. 2 Yuanmingyuan West Road, Beijing, 100193 China; 2 State Key Laboratory of Cotton Biology, Institute of Cotton Research of the Chinese Academy of Agricultural Sciences, Anyang, 455000 China

**Keywords:** Cuticle, *GhWIN2*, Jasmonic acid, Salicylic acid, Systems biology, VIGS, *Verticillium dahliae*

## Abstract

**Background:**

Metabolic pathways are interconnected and yet relatively independent. Genes involved in metabolic modules are required for the modules to run. Study of the relationships between genes and metabolic modules improves the understanding of metabolic pathways in plants. The WIN transcription factor activates the cuticle biosynthesis pathway and promotes cuticle biosynthesis. The relationship between the WIN transcription factor and other metabolic pathways is unknown. Our aim was to determine the relationships between the main genes involved in cuticle biosynthesis and those involved in other metabolic pathways. We did this by cloning a cotton *WIN* gene, *GhWIN2*, and studying its influence on other pathways.

**Results:**

As with other *WIN* genes, *GhWIN2* regulated expression of cuticle biosynthesis-related genes, and promoted cuticle formation. Silencing of *GhWIN2* resulted in enhanced resistance to *Verticillium dahliae*, caused by increased content of salicylic acid (SA). Moreover, silencing of *GhWIN2* suppressed expression of jasmonic acid (JA) biosynthesis-related genes and content. *GhWIN2* positively regulated the fatty acid biosynthesis pathway upstream of the JA biosynthesis pathway. Silencing of *GhWIN2* reduced the content of stearic acid, a JA biosynthesis precursor.

**Conclusions:**

*GhWIN2* not only regulated the cuticle biosynthesis pathway, but also positively influenced JA biosynthesis and negatively influenced SA biosynthesis.

**Electronic supplementary material:**

The online version of this article (10.1186/s12870-019-1888-6) contains supplementary material, which is available to authorized users.

## Background

Plants are constantly stimulated by environmental signals, some of which inhibit growth and development. Plants have developed many structures, such as the cuticle, that increase adaptation or tolerance to these stresses [1, 2]. The plant cuticle is a ubiquitous and chemically heterogeneous lipophilic layer composed of biopolymers, mainly comprising waxes, and cutin, a lipid polymer [3]. The cutin matrix consists mainly of esterified 16/18-carbon hydroxy and epoxy-hydroxy fatty acids (FAs) [4]. The waxes are formed by very-long-chain fatty acid (VLCFA) derivatives [5], and they cover or are embedded in the cutin matrix. The wax components are produced in three steps. First, 16/18-carbon long-chain acyl-coenzymeAs (C_16_/C_18_-acyl-CoAs) are produced from 16/18-carbon long-chain FAs that are catalyzed by long-chain acyl-CoA synthetases (LACSs) in the plastids of epidermal cells [6]. Second, the FAs are extended from C_16_/C_18_-acyl-CoAs to VLCFA-acyl-CoAs (>C_18_, with more than 18 C atoms), catalyzed by fatty acid elongases (FAEs) on the endoplasmic reticulum membrane [7]. FAE enzyme complexes consist of β-ketoacyl-CoA synthase (KCS), β-ketoacyl-CoA reductase (KCR), 3-hydroxyacyl-CoA dehydratase (HCD), and enoyl-CoA reductase (ECR) [8–12] Finally, VLCFA-acyl-CoAs further react to form wax components, mediating the alcohol- or alkane-forming pathways [7, 13–16]. Many genes encoding enzymes involved in these pathways have been studied.

*WIN1* (*wax inducer 1*) was first reported to transcriptionally activate epidermal wax biosynthesis in *Arabidopsis* [17]. The barley *WIN*/*SHN* gene, *Nud*, controls grain with adhering hulls by regulating a lipid biosynthesis pathway [18]. The tomato (*Solanum lycopersicum*) *SlWIN3/SHN3* gene regulates cuticle formation in fleshy fruits [19]. *WIN* transcription factors function redundantly to regulate the epidermal patterning of flower organs in *Arabidopsis* [13]. Most of the studies about *WIN* genes have focused on the regulation of cuticle biosynthesis. In addition, overexpression of an *Arabidopsis WIN* gene in rice activates cellulose biosynthesis and represses lignin biosynthesis [20]. Beyond that, there have been no reports about the influence of *WIN* on other metabolic pathways.

Jasmonic acid (JA) is an important plant hormone. It is needed for plant growth and development, survival under stress, and throughout the life-cycle. JA is biosynthesized from α-linolenic acid [21]. Linolenic acid is catalyzed by lipoxygenase (LOX) to produce 13-hydroperoxyoctadecatrienoic acid (13-HPOT) [22]. Stearic acid is converted to oleic acid, linoleic acid, and further to α-linolenic acid through a desaturation reaction [23]. Stearic acid has a role into the cuticle biosynthesis pathway [24]. The JA biosynthesis pathway competes with the cuticle biosynthesis pathway for precursors.

Salicylic acid (SA) is an important hormone that is involved in plant immune responses. It regulates the expression of many pathogenesis-related proteins (PRs) [25]. SA is involved in plant defense against *Verticillium dahliae* [26–29]. Two pathways of SA biosynthesis in plants have been reported. First, in *Arabidopsis*, SA appears to be synthesized primarily through an isochorismate-utilizing pathway. Second, phenylalanine forms a substrate in the SA biosynthesis pathway [30]. Both pathways begin with shikimic acid. Lignin is a phenolic heteropolymer that constitutes an important component of plant secondary cell walls, and shikimic acid is the precursor of lignin biosynthsis [31]. The shikimate pathway is responsible for the biosynthesis of tryptophan, tyrosine, and phenylalanine [32]. Phenylalanine is involved in the lignin pathway. *AtWIN1* negatively regulates lignin biosynthesis in transgenic rice plants [20]. The biosynthesis of lignin and SA share part of the same pathway [33]. Therefore, *WIN* has a role in lignin biosynthesis and may also influence SA accumulation.

Ideker [34] proposed the concept of systems biology in 2001. Briefly, systems biology is the study of living systems not only in terms of separate mechanistic and molecular-level components, but considering many components simultaneously [34, 35]. Metabolic pathways are interconnected, and yet they are relatively independent. For example, various hormone metabolic pathways exert their biological functions synergistically or antagonistically by forming complex and intersecting networks of regulatory pathways [36]. The WIN transcription factor positively regulates the cuticle biosynthesis pathway [17, 37]. Aside from its effects on lignin biosynthesis, it is unknown how the WIN transcription factor influences other metabolic pathways related to the cuticle biosynthesis pathway [20]. Here, we cloned a *WIN* gene, *GhWIN2*, from cotton (*Gossypium hirsutum*), and aimed to determine its role in the regulation of cuticle development and the influence on SA and JA accumulation.

## Results

### Characterization of GhWIN2

**Fig. 1:**
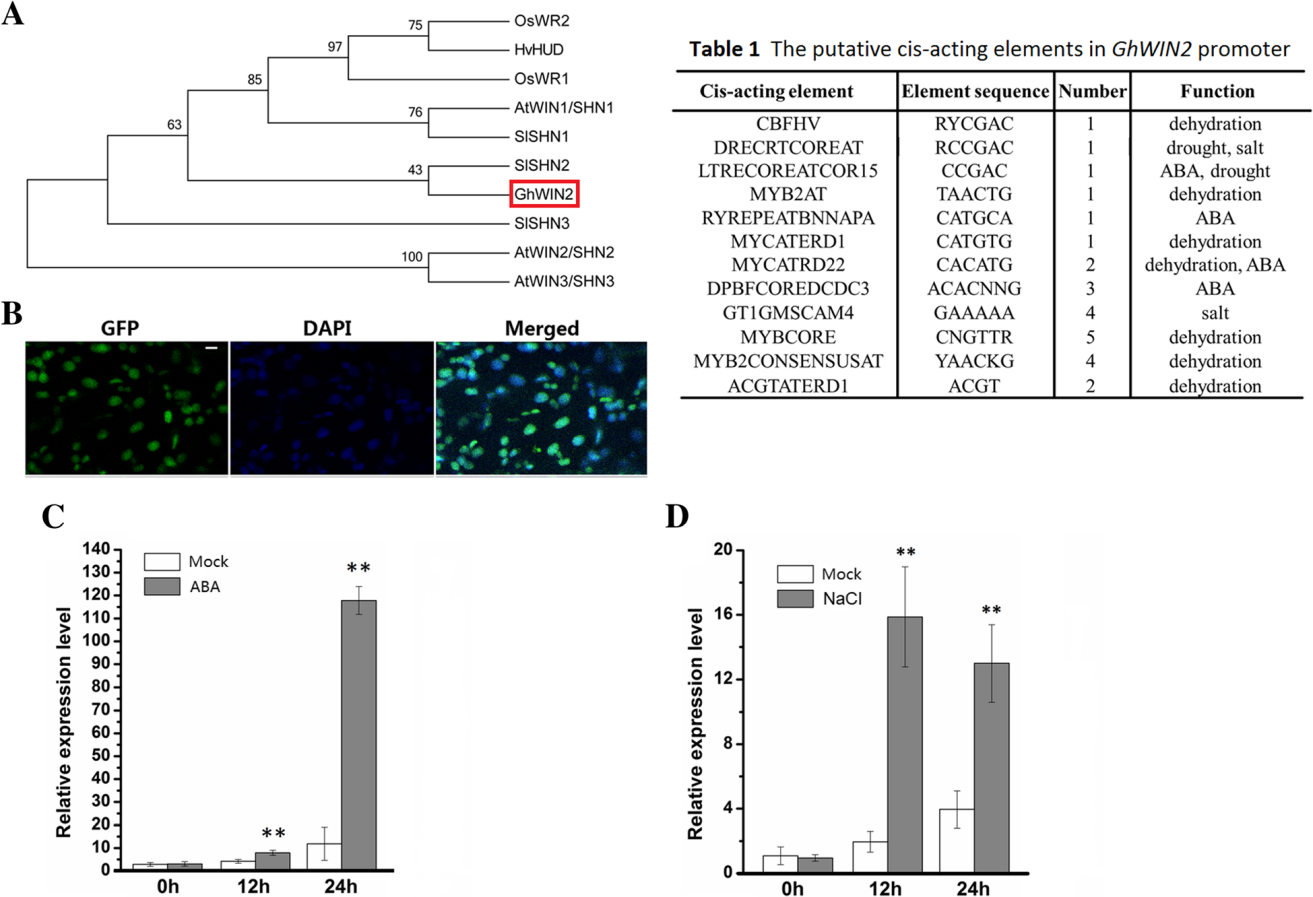
Cloning and characterization of *GhWIN2*. (**a**) Phylogenetic analysis of WIN proteins from cotton (*Gossypium hirsutum*, Gh), rice (*Oryza sativa*, Os), barley (*Hordeum vulgare*, Hv), tomato (*Solanum lycopersicum*, Sl), and *Arabidopsis* (At). Accession numbers: OsWR1(Os02g0202000); OsWR2(AK061163); HvHUD (KP245804.1); AtWIN1/SHN1(AT1g15360); AtWIN2/SHN2(At5g11190); AtWIN3/SHN3(At5g25390); SlSHN1(XP_004235965.1); SlSHN2(XP_004251719.1); SlSHN3(NM_001319202). The GhWIN2 is indicated by a red box. The rooted neighbor-joining tree was based on multiple sequence alignment using the MEGA5.1 software. (**b**) Subcellular localization of *GhWIN2*. GFP and DAPI fluorescence in the cotyledons of five-day-old plants of line 4 (scale bars: 10 μm). (C, D) The expression of *GhWIN2* in two-week-old cotton seedlings after treatment with 100 μM ABA (**c**) and 100 mM NaCl (**d**). The mock samples were treated with double distilled water. Data were from three independent replicates and the results are means ± SD. The relative transcription levels were normalized using *GhUBQ7*. Asterisks indicate significant differences between treated and mock plants according to Student’s *t*-test (* *P* < 0.05; ** *P* < 0.01)

Many *WIN*/*SHN* orthologs have been reported to affect various aspects in plant physiological processes [17, 37]. However, less is known about the cotton *WIN*/*SHN* orthologs. By using the full-length AtWIN1 amino acid sequence to perform a Blast query against the *G. hirsutum* genome database (https://cottonfgd.org/), we found eleven sequences of *WIN*/*SHN* orthologs in the cotton genome. Those sequences encode six putative amino acid sequences (Additional file 1: Figure S1). Next, primers were designed for full length amplification of those SHN orthologs. Unfortunately, we only cloned Gh_A07G036100 (XP_016720718) from two-week-old cotton seedlings. No expression of other homologous sequences was detected by RT-qPCR in seedings. Phylogenetic analysis revealed that this sequence was closely related to *SlSHN2*/*WIN2* (Fig. 1a) [19]. Thus, we named this gene *GhWIN2*.

We detected subcellular localization in the transgenic *Arabidopsis* plants that stably expressed *GhWIN2* (Additional file 1: Figure S2 and Figure S3). We selected transgenic *Arabidopsis* line 4, which had the highest expression levels among the transgenic lines, for analyzing the subcellular localization. DAPI staining was used to stain the nucleus. GhWIN2-GFP and DAPI fluorescence were colocalized in the nucleus (Fig. 1b).

To explore the expression pattern of *GhWIN2*, we analyzed the *GhWIN2* promoter sequence obtained from the *G. hirsutum* genome database (Table 1). This promoter sequence contains cis-acting elements that are involved in ABA and drought response. The expression of *GhWIN2* was strongly induced by abscisic acid (ABA) and sodium chloride (NaCl) treatment in cotton seedlings (Fig. 1c and d).

### GhWIN2 activates the cuticle biosynthesis pathway

**Fig. 2:**
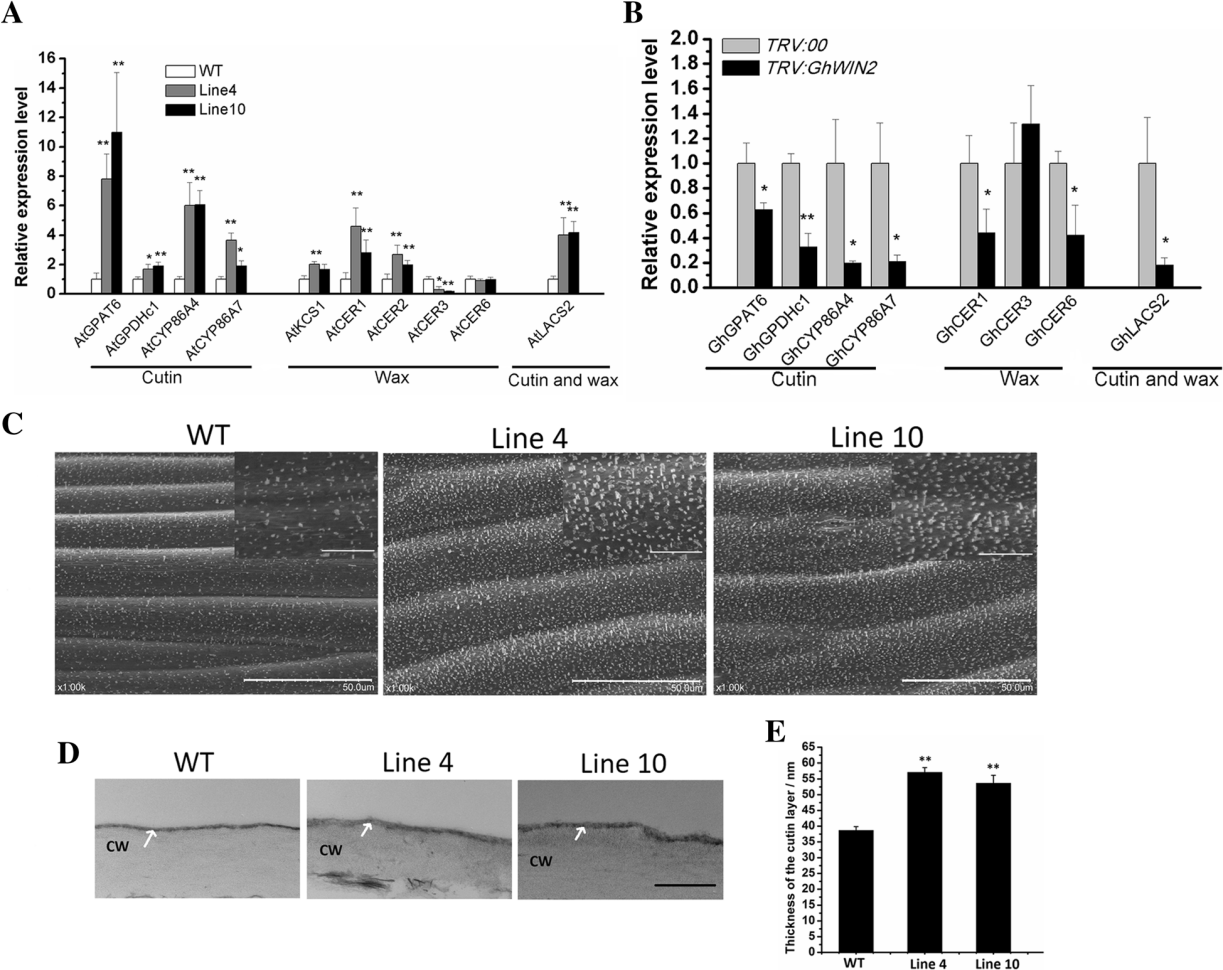
*GhWIN2* regulation of cuticle development. (**a**) Expression of genes involved in cuticle biosynthesis in four-week-old WT and transgenic *Arabidopsis* plants. *Arabidopsis elongation factor-1-α* gene (*EF-1-α*) was the endogenous reference for normalization. Data represent means ± SD for three biological replicates. Student’s *t*-test; * *P* < 0.05, ** *P* < 0.01. (**b**) Expression of the putative cuticle biosynthesis-related genes in cotton plants 14 days after infiltration. (**c**) SEM images of the stems of the WT and transgenic *Arabidopsis* plants. (Scale bars in main image, 50 μm; scale bars in insets, 10 μm) (**d**) TEM images of the cuticle of the upper leaf surface (scale bar: 500 nm). CW, cell wall; white arrows mark the cuticle. Images were taken at 50,000× magnification. (**e**) The thickness of the cuticle layer from the leaf upper surface (**d**). Data are presented as means ± SD from at least four independent biological replicates. Asterisks denote Student’s *t* test significance compared with WT plants (* *P* < 0.05; ** *P* < 0.01)

To examine the functional similarity between *GhWIN2* and other known *WINs*, we detected the expression level of several cuticle-related genes in the wild-type (WT) and transgenic *Arabidopsis* plants (lines 4 and 10). The selection of those genes was based on previous studies in which the WIN proteins that regulate genes were characterized [17, 38, 39]. The expression of the detected cutin biosynthesis-related genes *AtGPAT6* (encoding glycerol-3-phosphate acyltransferase 6), *AtGPDHc1* (encoding cytosolic G-3-P dehydrogenase), *AtCYP86A4* (encoding cytochrome P450 enzymes), and *AtCYP86A7* was greater in lines 4 and 10 than in the WT (Fig. 2a). Expression of the wax biosynthesis-related genes *AtKCS1* (encoding 3-ketoacyl-CoA synthase 1), *AtCER1* (encoding ECERIFERUM1), and *AtCER2* (ECERIFERUM2) was greater in lines 4 and 10, whereas that of *AtCER3* (ECERIFERUM3) was less, and that of *AtCER6* (ECERIFERUM6) was unchanged, relative to expression in the WT. Expression of the cutin and wax biosynthesis-related gene *AtLACS2* (encodes long-chain acyl-coenzyme A synthetase) was greater in lines 4 and 10 than in the WT.

We detected expression levels of the putative cotton orthologs of known *Arabidopsis* cuticle biosynthesis-related genes in *GhWIN2* silenced in cotton plants (hereafter referred to as “*TRV:GhWIN2* plants”; Additional file 1: Figure S4 and Figure S5). Expression of the detected cutin biosynthesis-related genes *GhGPAT6*, *GhGPDHc1*, *GhCYP86A4*, and *GhCYP86A7* was lower in *TRV:GhWIN2* plants than in the *TRV:00*. Expression of wax biosynthesis-related genes *GhCER1* and *GhCER6* were lower, whereas that of *GhCER3* was unchanged, relative to expression in the *TRV:00*. Expression of *GhLACS2* was also lower in *TRV:GhWIN2* plants than in the *TRV:00* plants (Fig. 2b). Overall, *GhWIN2* up-regulated the expression of the most cuticle biosynthesis-related genes in plants.

Scanning electron microscopy revealed that the transgenic *Arabidopsis* plants had greater wax crystal accumulation in the stem and a thicker abaxial cuticle layer in the leaf than the WT plants (Fig. 2c, d, and e). These results indicate that *GhWIN2* activates the cuticle biosynthesis pathway.

### GhCYP86A4 is the target gene of GhWIN2

**Fig. 3:**
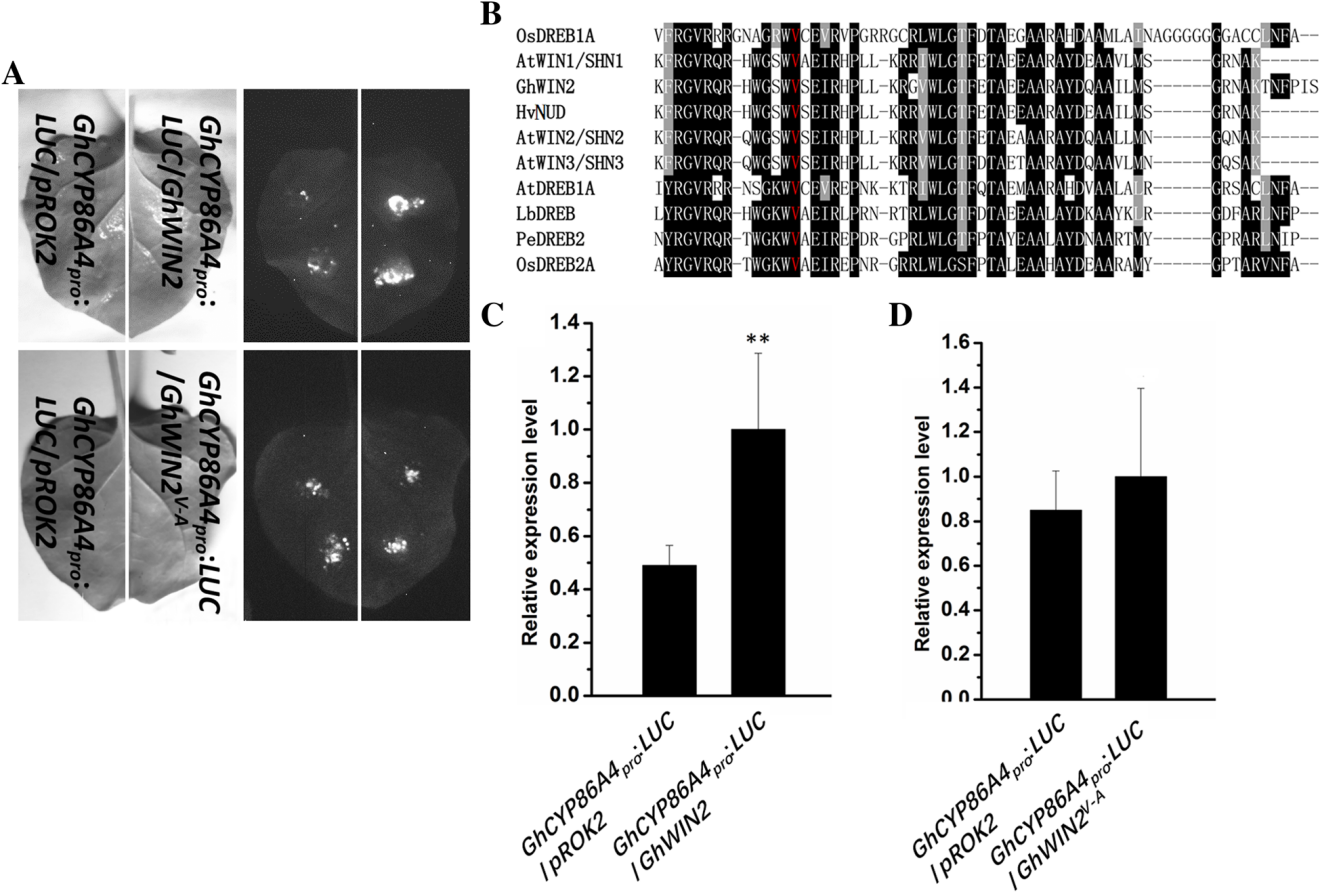
*GhWIN2* activation of the promoter of the *GhCYP86A4* gene. (**a**) Luminescence signal of transient co-expression of *GhWIN2*/*GhCYP86A4*_*pro*_:*LUC* or *GhWIN2*^*V-A*^/*GhCYP86A4*_*pro*_:*LUC* on *Nicotiana benthamiana* leaves. The photos were taken 2 days after infiltration with the same amount of *Agrobacterium tumefaciens* cultures containing the specified constructs. Two sites were injected for every treatment. (**b**) Sequence alignments of AP2/EREBP proteins. The conserved valine is marked in red. GenBank accession numbers: *Oryza sativa* OsDREB1A (AAN02486), OsDREB2A (AAN02487), AtDREB1A (Q9M0L0), *Limonium bicolor* LbDREB (ACP18975), *Populus euphratica* PeDREB2 (ABL86587), *Hordeum vulgare* HvNUD (AKF40403). (**c**, **d**) Expression of *LUC* transcripts 2 days after co-infiltration of *Agrobacterium* cells harboring *GhWIN2*/*GhCYP86A4*_*pro*_:*LUC* (**c**) and *GhWIN2*^*V-A*^/*GhCYP86A4*_*pro*_:*LUC* (**d**). Data represent means ± SD from three independent biological replicates; Student’s *t*-test; * *P* < 0.05, ** *P* < 0.01

To explore the role of *GhWIN2* in transcriptional activation, we cloned the promoters of *GhLACS2*, *GhCYP86A4*, and *GhCYP86A7* genes. Using an in vivo transient gene expression assay, we found that only co-infiltration of *35S:GhWIN2* and *GhCYP86A4*_*pro*_-*LUC* constructs resulted in transcriptional activation (Fig. 3a). Dehydration-responsive element-binding (DREB) and WIN transcriptional factors all belong to the subgroup of AP2/EREBP family that contains an AP2/EREBP domain involved in DNA-binding. Sequence alignment showed that GhWIN2 shares significant sequence identity with other AP2/EREBP proteins at the AP2/EREBP domain (Fig. 3b). From previous studies of DREB proteins, the single amino acid substitution of valine to alanine was sufficient to nullify the interaction between protein and DNA [40–42]. The corresponding residues are conserved in WIN proteins (Fig. 3b). Thus, we generated *GhWIN2*^*V-A*^ with alanine substitution at the corresponding position. Co-infiltration of *35S:GhWIN2*^*V19A*^ and *GhCYP86A4*_*pro*_-*LUC* constructs nullified the transcriptional activation (Fig. 3a). In addition, qPCR analysis showed that co-infiltration of *35S:GhWIN2* and *GhCYP86A4*_*pro*_*-LUC* activated the expression levels of the *LUC* gene, while co-infiltration of *35S:GhWIN2*^*V19A*^ and *GhCYP86A4*_*pro*_-*LUC* did not (Fig. 3c and d).

### ABA regulates the expression of cuticle biosynthesis genes mediated by GhWIN2

**Fig. 4:**
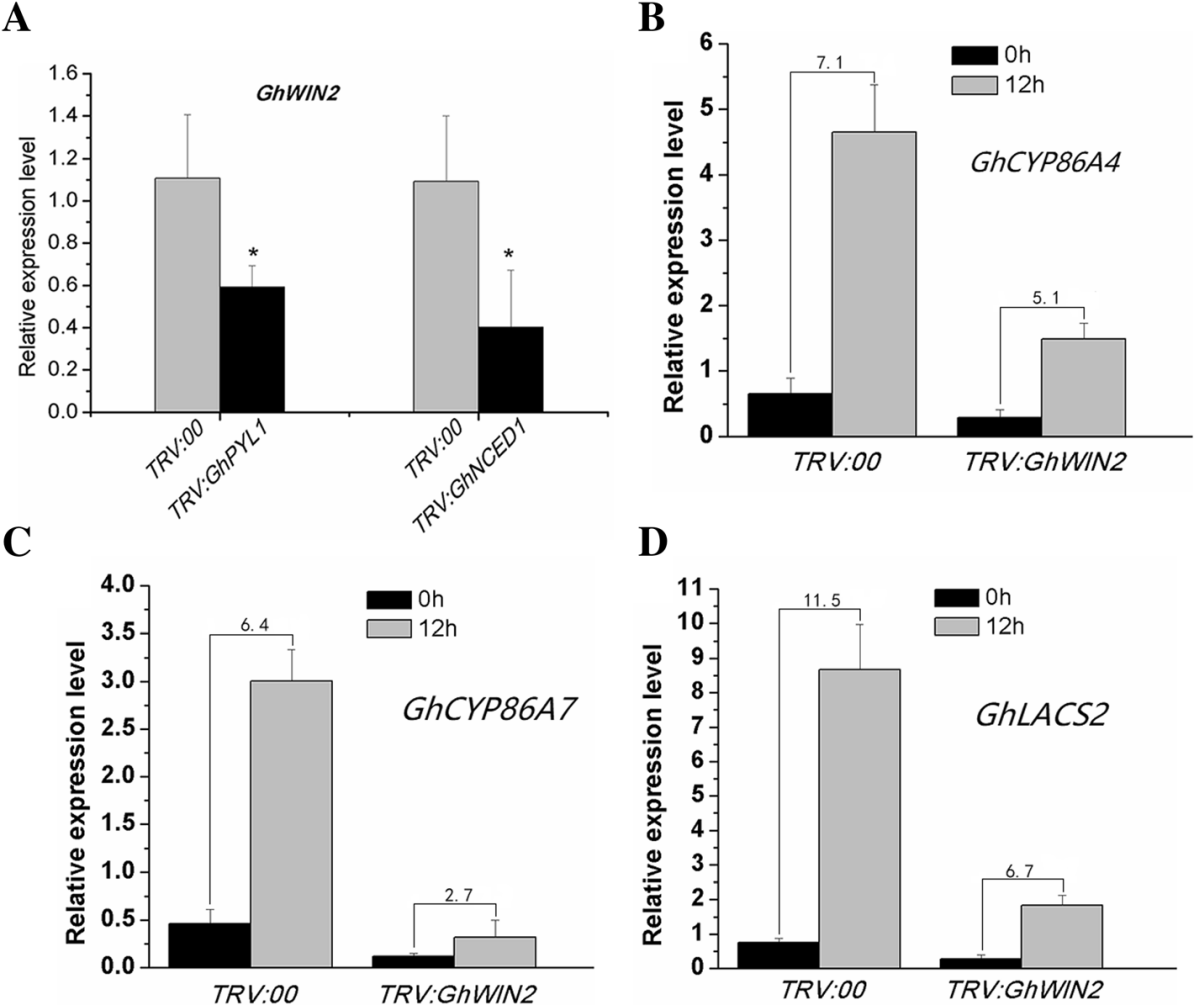
*GhWIN2* involvement in the ABA-cuticle pathway. (**a**) Expression levels of *GhWIN2* were detected in *TRV:GhPYL1* and *TRV:GhNCED1* plants 14 days after infiltration. Values represent the means ± SD from at least three independent biological replicates. Student’s *t*-test; * *P* < 0.05. (B–D) Expression levels of *GhCYP86A4* (**b**), *GhCYP86A7* (**c**), and *GhLACS2* (**d**) in *TRV:00* and *TRV:GhWIN2* plants after treatment with 100 μM ABA. The numbers represent the inducible multiples of plants treated with ABA compared to untreated plants

We found that exogenous ABA strongly induced the expression of *GhWIN2* (Fig. 1c). To further study the relationship between ABA and *GhWIN2*, we silenced *GhPYL1* and *GhNCED1*, two key ABA biosynthesis genes in cotton [43, 44], and detected the expression levels of *GhWIN2* in *TRV:GhPYL1* and *TRV:GhNCED1* plants (Additional file 1: Figure S6). Expression of *GhWIN2* in *TRV:GhPYL1* and *TRV:GhNCED1* plants was suppressed after silencing of *GhPYL1* and *GhNCED1* (Fig. 4a).

To further identify the role of *GhWIN2* in ABA-cuticle pathway, expression levels of *GhCYP86A4*, *GhCYP86A7*, and *GhLACS2* was detected in *TRV:00* and *TRV:GhWIN2* plants. We found that exogenous ABA induced expression of these three genes in *TRV:00* and *TRV:GhWIN2* plants, whereas the inducible multiple was lower in *TRV:GhWIN2* plants (Fig. 4b-d). These results suggest that ABA induces the expression of cuticle-related genes mediated by *GhWIN2*.

### GhWIN2 negatively regulates plant resistance to fungal pathogens

**Fig. 5:**
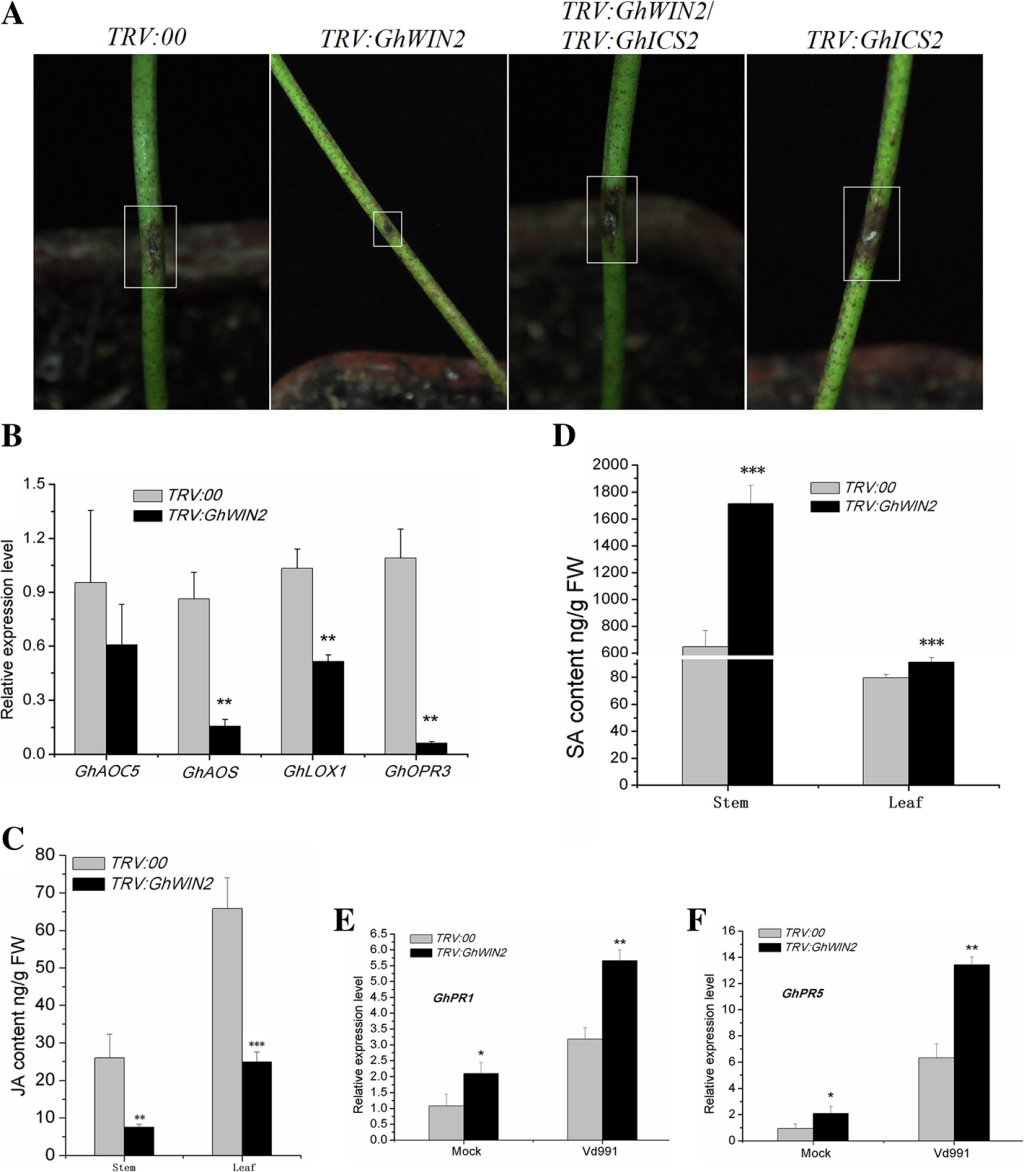
*GhWIN2* negative regulation of plant immune response to *V. dahliae*. (**a**) Disease symptoms after inoculation with *V. dahliae*. (**b**) Expression of JA biosynthesis-related genes in *TRV:00* and *TRV:GhWIN2* plants 14 days after agroinfiltration. Values are shown as means ± SD from at least three independent biological replicates. Student’s *t*-test; * *P* < 0.05. (C, D) Content of JA (**c**) and SA (**d**). Values are the means ± SD from six independent biological replicates. Student’s *t*-test; * *P* < 0.05, ** *P* < 0.01, *** *P* < 0.001. (**e**, **f**) Expression of marker genes involved in SA response

Previous studies have shown that WIN orthologs positively or negatively regulate plant immune responses, depending on the plant species studied [19, 38]. Here, we were interested in finding out how *GhWIN2* functions in cotton immune response. To examine this, we challenged *TRV:GhWIN2* and *TRV:00* plants with *V. dahliae*. We detected greater resistance to *V. dahliae* in *TRV:GhWIN2* plants than in the *TRV:00* plants (Fig. 5a). An analysis of relative fungal biomass showed that there was less fungus in *TRV:GhWIN2* plants (Additional file 1: Figure S7). JA is involved in plant immune response to *V. dahliae* [45, 46]. Surprisingly, expression of the putative or identified JA biosynthesis-related genes was suppressed and content of JA decreased in *TRV:GhWIN2* plants (Fig. 5b and c). However, the content of SA and expression of the SA-responsive genes *GhPR1* and *GhPR5* was significantly greater in *TRV:GhWIN2* plants than in *TRV:00* plants (Fig. 5d and e). To determine whether SA enhanced the immune response, we decreased the content of SA by silencing the putative SA biosynthesis gene *GhICS2* (*TRV:GhICS2*) [47]. Challenging with *V. dahliae* resulted in lower SA content in the *TRV:GhICS2* plants and the *TRV:GhWIN2*/*TRV:GhICS2* two-gene-silenced plants than in the *TRV:00* and *TRV:GhWIN2* plants (Additional file 1: Figure S8). Resistance to *V. dahliae* was lower in the two-gene-silenced plants than in the *TRV:GhWIN2* plants (Fig. 5a; Additional file 1: Fig. S7). These results indicate that SA enhanced resistance to *V. dahliae* in *TRV:GhWIN2* plants.

**Fig. 6:**
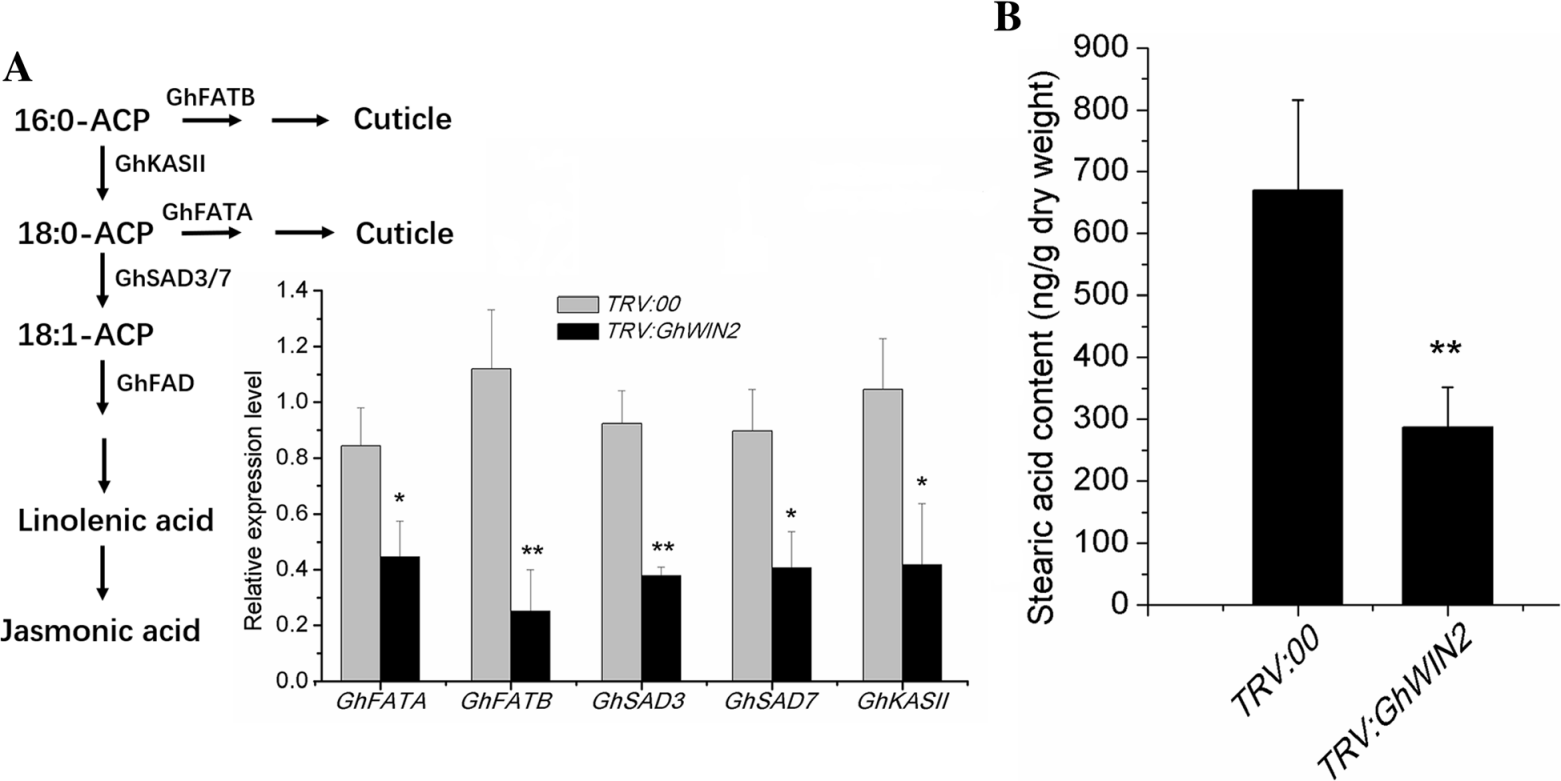
Regulation of 16:0-ACP metabolic flux. (**a**) *GhWIN2* positively regulates the expression of 16:0-ACP metabolic flux-related genes. Values are shown as means ± SD from three independent biological replicates. Student’s *t*-test; * *P* < 0.05, ** *P* < 0.01. (**b**) Content of stearic acid in *TRV:00* and *TRV:GhWIN2* plants

Next, we detected expression of genes involved in the fatty acid biosynthesis pathway upstream of the cuticle and JA biosynthesis pathway, and assessed the stearic acid content (Fig. 6). We found that expression levels of *GhFATA*, *GhFATB*, *GhSAD3*, *GhSAD7*, and *GhKASII* [48–51] were lower in *TRV:GhWIN2* plants than in *TRV:00* plants. Content of the stearic acid was also lower in *TRV:GhWIN2* plants than in *TRV:00* plants.

## Discussion

The aerial parts of land plants are covered by a cuticle, a hydrophobic layer that prevents the epidermal cells from having direct contact with the environment [7]. Many studies about the cuticle have been reported. However, the relationship between the cuticle biosynthesis pathway and other metabolic pathways has rarely been reported. To obtain deeper insight into the cuticle biosynthesis pathway, we cloned *GhWIN2* from cotton. We have identified *GhWIN2* as a positive regulator of the cuticle synthesis pathway. Our results provide evidence that reduced expression of *GhWIN2* negatively regulates JA accumulation and positively regulates SA accumulation, and further confers resistance against *V. dahliae.*

It is known that WIN transcription factors transcriptionally activate the expression of cuticle synthesis-related genes [13, 52], resulting in cuticle accumulation. In barley, the *Nud* gene, which is homologous to the *Arabidopsis WIN1*/*SHN1*, is responsible for the control of a lipid biosynthesis pathway, generating organ adhesion [18]. We found that GhWIN2 belongs to the WIN protein family; first, it is localized at the nucleus (Fig. 1b); second, its overexpression in *Arabidopsis* increased the amount of wax crystals on the stem and thickness of abaxial cuticle in the leaf (Fig. 2c, d, and e); third, it transcriptionally regulated the expression of cuticle-related genes (Fig. 2a and b; Fig. 3).

Previous studies have shown that WIN orthologs transcriptionally activate target genes [19, 37, 53]. Here, to explore the role of *GhWIN2* in transcriptional activation, we cloned the promoters of *GhLACS2*, *GhCYP86A4*, and *GhCYP86A7* genes; we selected these genes based on previous studies in which WIN orthologs target genes were identified [19, 37, 53]. We found that GhWIN2 only activates the transcription of *GhCYP86A4* promoter (Fig. 3a).

The DREB/ERF-type transcription factor belongs to the AP2/EREBP family [40]. DREB proteins contain two conserved amino acids in the AP2/EREBP domain, of which valine is especially important for DNA-binding [40–42]. Consistent with those findings, we found that GhWIN2 is a subfamily in the AP2/EREBP family, and contains a conserved AP2/EREBP domain (Fig. 3b). These analyses point to the potential role of *GhWIN2* in transcriptional activation of the corresponding conserved valine. We observed that the single amino acid substitution of valine to alanine was sufficient to nullify the transcriptional activation of GhWIN2 (Fig. 3a).

In *Arabidopsis*, it has been shown that the expression of many cuticle-related genes is suppressed or induced in ABA biosynthesis mutants, depending on the mutant used and the genes studied [54]. Exogenous ABA inhibits expression of cuticle biosynthesis-related genes in *Physcomitrella patens*, whereas those orthologs were induced in *Arabidopsis* [54]. These results led us to detect how ABA regulates expression of cuticle-related genes in cotton. Here, we found that exogenous ABA strongly induced expression of *GhWIN2* (Fig. 1c). This is consistent with previous reports that *TdWIN1* was strongly induced by ABA in wheat [55]. In addition, expression of *GhWIN2* was suppressed in *TRV:GhPYL1* and *TRV:GhNCED1* plants (Fig. 4a). These results indicate that GhWIN2 is an ABA-responsive transcription factor. In *Arabidopsis*, the genes *MYB16*, *MYB94*, *MYB96* and *DEWAX* are involved in the ABA-cuticle regulatory pathway [54]. There have been no reports that *AtWIN1/SHN1* is involved in ABA-cuticle pathway. Here, we found that ABA induced some cuticle biosynthesis genes and that this was mediated, or partially mediated, by *GhWIN2* (Fig. 4b-d). Thus, the mechanism of cuticle biosynthesis regulated by ABA is conserved and sophisticated in various species. As previously reported, although ABA generally induces expression of the transcription factors that positive regulate of cuticle biosynthesis, it also suppresses expression of one of these positive regulators, HDG1 [54].

**Fig. 7:**
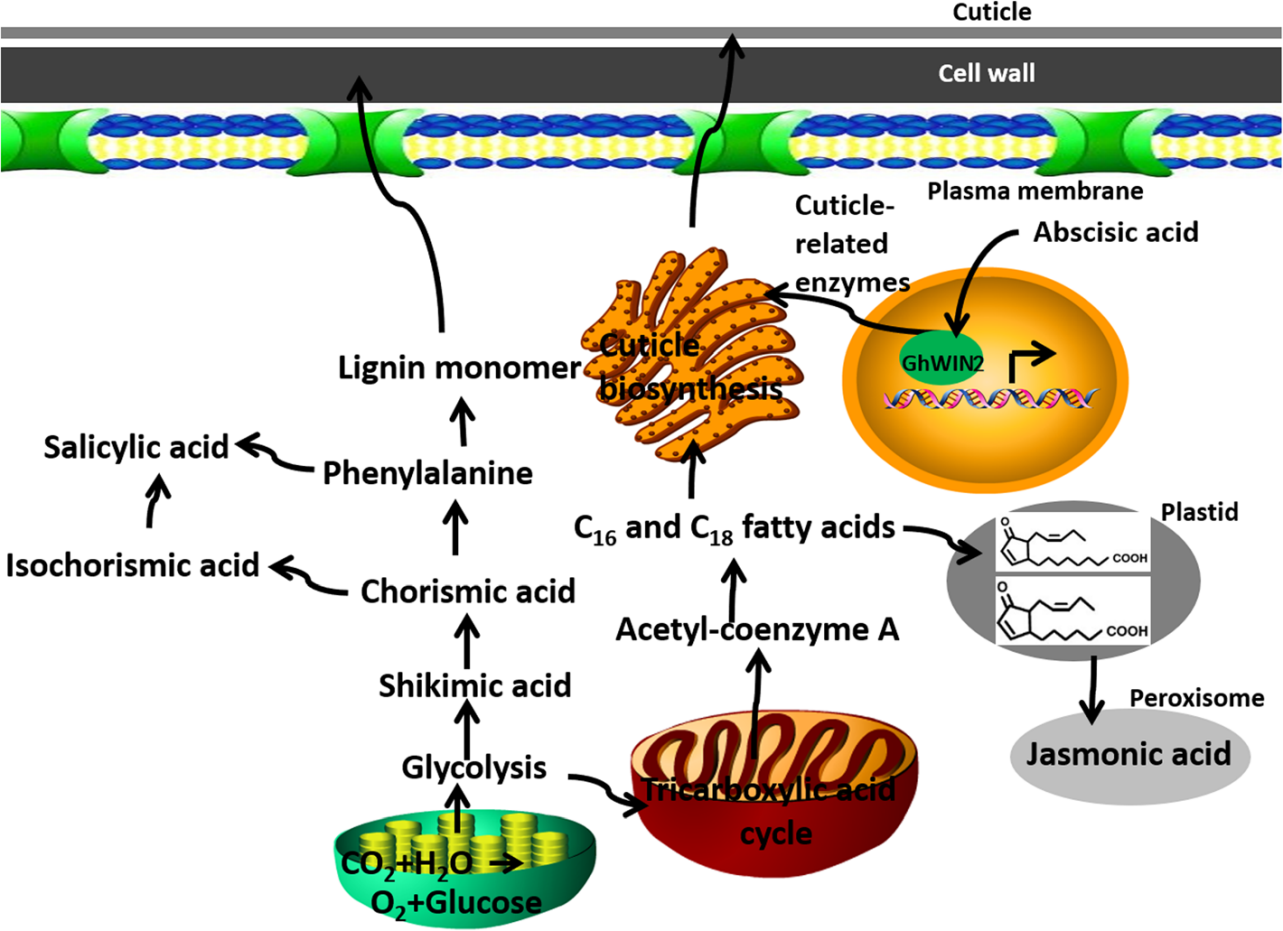
Model of *GhWIN2*-related metabolic pathways. *GhWIN2* positively regulated cuticle biosynthesis and content of JA, and negatively affected SA biosynthesis

Previous studies have shown that WIN orthologs positively or negatively regulate immune responses, depending on the plant and pathogen species studied. In *Arabidopsis*, overexpression of *AtWIN1*/*SHN1* caused reduced expression of *PDF1.2*, which compromised resistance to *Botrytis cinerea* and *Alternaria brassicicola* [56]. However, tomato *SlWIN3*/*SHN3* conferred resistance in fruit against the fungus *Colletotrichum coccodes*, by causing a thickened cuticle that prevents fungal penetration [19]. Here, we found that *GhWIN2* conferred sensitivity to *V. dahliae* (Fig. 5a). The thickened cuticle may contribute to the increased immune response, but its efficacy against *V. dahliae* was not great. Expression of *PDF1.2* in *AtWIN1*/*SHN1* overexpressed *Arabidopsis* plants was dramatically lower than in wild-type *Arabidopsis* plants [56]. In addition, the JA and cuticle biosynthesis pathways share the same precursor, stearic acid [57–60]. Therefore, reduced expression of *GhWIN2* may lead to increased biosynthesis of JA, and this could explain why we the detected expression of JA biosynthesis-related genes (Fig. 7). Expression of putative or identified JA biosynthesis-related genes and content of JA was lower in *TRV:GhWIN2* plants than in *TRV:00* plants (Fig. 5b and c), probably because that the precursor of JA biosynthesis reduced caused by decreased expression of *GhWIN2*. Thus, *GhWIN2* may regulate the fatty acid biosynthesis pathway upstream of the cuticle biosynthesis pathway and the JA biosynthesis pathway simultaneously. As we predicted, the expression of genes related to stearic acid biosynthesis and stearic acid metabolism was reduced in *TRV:GhWIN2* plants (Fig. 6). These results indicated that *GhWIN2* regulated not only the cuticle biosynthesis pathway, but also the upstream pathways.

Content of SA and SA-responsive marker genes was substantially lower in *TRV:GhWIN2* plants than in *TRV:00* plants (Fig. 5d and e). This is consistent with the finding that overexpression of *AtWIN1*/*SHN1* in rice caused a 45% reduction in lignin content [20]. Biosynthesis of SA and lignin share part of the same pathway, and shikimic acid is a precursor to SA and lignin (Fig. 7). Thus, it is likely that WIN regulates biosynthesis of SA and lignin via the same mechanism. This is probably because abnormal expression of *GhWIN2* alters metabolic flux redirection. The greater resistance to *V. dahliae* that we observed in the *TRV:GhWIN2* plants was probably caused by the higher SA content in these plants. To evaluate this, we subjected the putative gene *GhICS2* involved in SA biosynthesis to functional analysis [47]. VIGS constructs for *GhWIN2* and *GhICS2* were used together to generate two-gene silenced plants, *TRV:GhWIN2*/*TRV:GhICS2*. Content of SA and resistance to *V. dahliae* was lower in *TRV:GhWIN2*/*TRV:GhICS2* plants than in *TRV:GhWIN2* plants. Thus, we conclude that SA conferred resistance to *V. dahliae* in *TRV:GhWIN2* plants.

We obtained eleven sequences of *WIN*/*SHN* orthologs in the cotton genome. However, we only cloned *GhWIN2* from cotton seedings and did not detect the expression of other sequences, indicating that other orthologs may not be expressed at the seedling stage, or it may be just that we have not detected them. If the latter, silencing of *GhWIN2* could have silenced other orthologs simultaneously considering their high homology. Thus, it is worth noting that the observed phenotypes may be the results of silencing of *WIN*/*SHN* orthologs.

## Conclusions

From the point of view of systems biology, there are intricate connections among different metabolic pathways: they compete for substrates, have different metabolic kinetics, and their products can activate or inhibit other pathways [35]. Studying the connections between different metabolic pathways in plants is important in bioenergy and synthetic biology, and can expand our understanding of whole plant systems. The cuticle is related to plant drought tolerance and disease resistance. It acts as a barrier limiting non-stomatal water loss [4, 61, 62]. Few studies have examined the relationship between the cuticle biosynthesis pathway and other metabolic pathways. Here, we have provided evidence that *GhWIN2* not only regulates cuticle biosynthesis pathway, but also positively influences JA biosynthesis and negatively influences SA biosynthesis. The other *WIN*/*SHN* orthologs may also be involved in regulation of these physiological processes.

## Methods

### Plant and fungal cultivation

The state cotton variety 2,006,001 (original strain no. GK44) was kindly provided by the Cotton Research Institute, Chinese Academy of Agricultural Sciences. Germination was accelerated before sowing in soil. The cotton seedlings were grown for 2 weeks at day/night temperatures of 26 °C/23 °C in an incubator, using a 16 h light/8 h dark photoperiod cycle.

*Arabidopsis thaliana* Col-0 wild type (WT) and transgenic *Arabidopsis* plants were grown in soil in an incubator at 23 °C, 70% relative humidity, with a 16 h light / 8 h dark photoperiod. Seedlings were grown on agar plates containing 1% sucrose and 0.8% agar. Seeds were sterilized before being planted on the plate.

*Verticillium dahliae* strain Vd991 was cultured on a potato dextrose agar plate for 7 days at 26 °C and then inoculated into Czapek medium for 1 week. The spore suspension (10^6^ spores ml^− 1^) was then prepared by filtration.

### RNA extraction and RT-qPCR

Total RNA was extracted from the transgenic *Arabidopsis* plants or treated cotton plants using a plant RNA extraction kit (Biomed). Two micrograms of total RNA were reverse transcribed using a Fast Quant cDNA Reverse Kit (Tiangen Biotech Co., Ltd., Beijing, China). RT-qPCR was carried out using a SYBR® Premix Ex Taq (Tli RNaseH Plus) (Takara, Shiga, Japan). The endogenous genes *GhUBQ7* (DQ116441) and *EF-1-α* were used as the control in cotton and *Arabidopsis* plants, respectively. Reactions were amplified on an ABI7500 thermocycler (Applied Biosystems, Foster City, CA, USA). The transcription levels of *GhWIN2* were analyzed by the comparative CT (2^-△△CT^) method. Relative fungal biomass was detected by RT-qPCR. The *V. dahliae* specific primers, ITS1-F/ST-Ve1-R, were used (Additional file 1: Table S1).

### Subcellular localization

To obtain *GhWIN2*-overexpressing *Arabidopsis* plants, *GhWIN2* was amplified using the primers WIN-1300-F/WIN-1300-R (Additional file 1: Table S1), with *Pst1* and *Spe1* cleavage sites at the 5′ and 3′ ends, respectively. Next, the sequence was inserted into a modified Super-pCAMBIA1300 vector (Additional file 1: Figure S2). The recombinant construct was transformed into *Agrobacterium tumefaciens* strain GV3101. GV3101 was then used to infect *Arabidopsis thaliana* to obtain transgenic plants [63].

Seedlings were grown for five days under standard conditions. To stain nuclei, five day-old transgenic seedlings were submerged in PBS containing 5 μg/mL 4′-6-diamidino-2-phenylindole (DAPI) (Sigma) and then incubated for 15 min before imaging [64]. Imaging of GFP and DAPI was performed with a FLUOVIEW FV1000 confocal laser scanning microscope (Olympus, Tokyo, Japan). GFP and DAPI were excited at 405 nm and 488 nm, respectively.

### Transient gene expression assay

The promoter sequence of *GhCYP86A4* was cloned using primers GhCYP86A4_pro_-F/GhCYP86A4_pro_-R (Additional file 1: Table S1). The Gateway cloning system (Invitrogen) was used. The promoter sequence was cloned into vector pGWB435, which contains an *LUC* reporter gene [65]. The construct *GhCYP86A4*_*pro*_:*LUC* was then transformed into *Agrobacterium tumefaciens* strain GV3101. The GV3101 was cultured in LB medium containing 50 mg/mL spectinomycin and 50 mg/mL rifampicin. The coding sequence of *GhWIN2* and *GhWIN2*^V-A^ was cloned into the vector pROK2 to generate *35S:GhWIN2* and *35S:GhWIN2*^V-A^. These two constructs were then transformed into *Agrobacterium tumefaciens* strain GV3101. GV3101 harboring *35S:GhWIN2* or *35S:GhWIN2*^V-A^ was then cultured in LB medium containing 50 mg/mL kanamycin and 50 mg/mL rifampicin. For transient expression assay in tobacco, the *Agrobacterium tumefaciens* strain GV3101 cells containing *GhCYP86A4*_*pro*_:*LUC* and *35S:GhWIN2*/*35S:GhWIN2*^V-A^ were mixed and treated with infiltration buffer (200 mM acetosyringone, 10 mM MES, pH 5.6; 10 mM MgCl_2_) for 3 h. Next, the mixed cultures were injected into the leaf using a needleless syringe. The plants were then grown in the dark for 24 h and then under normal growth conditions for 48 h. The LUC signal was measured using a CCD camera (1300B; Roper) after being sprayed with 1 mM luciferin (Sigma-Aldrich).

### Virus-induced gene silencing

The fragments of *GhWIN2* was amplified and cloned into an improved *pTRV2* virus-induced gene silencing (VIGS) vector, pYL192 [65–67]. The recombinant plasmid *pTRV2:GhWIN2* was then transformed into *Agrobacterium tumefaciens* strain GV3101. Gene silencing was conducted according to the method described in Li et al. [65]. *Agrobacterium* cultures harboring *pTRV1*, *pTRV2*, or *pTRV2:GhWIN2* were grown in LB medium containing 50 μg/mL rifampicin, 50 μg/mL kanamycin, 20 μM acetosyringone, and 10 mM MES until the OD_600_ reached a value of 1. The cotyledons of 14-day-old cotton seedlings were injected with a mixture of the *Agrobacterium* cultures harboring the *pTRV1*/*pTRV2* plasmids (1:1 ratio, v/v) as the control and *pTRV1*/*pTRV2:GhWIN2* plasmids (1:1 ratio, v/v) as the experimental group. We also constructed *pTRV2:GhCLA1* (*Cloroplastos alterados 1*; 500 bp) to detect the efficiency of silencing under our experimental conditions.

### Accession numbers

AtGPAT6, AT2G38110; AtGPDHc1, AT2G41540; AtCYP86A4, AT1G01600; AtCYP86A7, AT1G63710; AtKCS1, AT1G01120; AtCER1, At1G02205; AtCER2, At4G24510; AtCER3, At5g57800; AtCER6, At1g68530; AtLACS2, At1G49430; GhGPAT6, ADK23938.1; GhGPDHc1, XP_016671566.1; GhCYP86A4, XM_016840837.1; GhCYP86A7, XP_016719401.1; GhCER1, XP_016681695.1; GhCER3, XP_016725538.1; GhCER6, KT625616.1; GhLACS2, XP_016707966.1; GhAOC5, KF383427.1; GhAOS, ALG62633.1; GhLOX1, AF361893.4; GhOPR3, NP_001313917.1; GhFATA, XP_016727762.1; GhFATB, XP_016720478.1; GhSAD3, XM_016885870.1; GhSAD7, XM_016843547.1; GhKASII, HM236494.1; GhPYL1, XM_016815548.1; GhNCED1, HM014161.

## Additional file

Additional file 1: Supplementary information. **Figure S1** Amino acid sequence alignment of cotton WIN orthologs. **Figure S2** Schematic representation of the plant expression vector pCAMBIA1300-*GhWIN2*. **Figure S3** Relative expression of *GhWIN2* in transgenic *Arabidopsis* plants. **Figure S4** Expression of *GhWIN2* in control and silenced cottons. **Figure S5** VIGS-mediated silencing of *GhCLA1* in cotton. **Figure S6** Relative expression of *GhPYL1* and *GhNCED1* in cotton plants 14 days after agroinfiltration. **Figure S7** Relative biomass of *V. dahliae* in infected cotton plants. **Figure S8** VIGS-mediated silencing of *GhICS2* in cotton. **Table S1** Primers used in this study. (PDF 1037 kb)

## Data Availability

The data sets during the current study are available from the corresponding author.

## Abbreviations

13-HPOT: 13-hydroperoxyoctadecatrienoic acid
ABA: Abscisic acid
C16/C18-acyl-CoA: 16/18-carbon long-chain acyl-coenzyme A
CER1: ECERIFERUM1
CER2: ECERIFERUM2
CER3: ECERIFERUM3
CER6: ECERIFERUM6
CYP: Cytochrome P450 enzymes
DREB: Dehydration-responsive element-binding
ECR: Enoyl-CoA reductase
FAEs: Fatty acid elongases
GPAT6: Glycerol-3-phosphate acyltransferase 6
GPDHc1: Cytosolic G-3-P dehydrogenase
HCD: 3-hydroxyacyl-CoA dehydratase
JA: jasmonic acid
KCR: β-ketoacyl-CoA reductase
KCS: β-ketoacyl-CoA synthase
KCS1: 3-ketoacyl-CoA synthase 1
LACS2: Long-chain acyl-coenzyme A synthetase
LACSs: Long-chain acyl-CoA synthetases
LOX: lipoxygenase
NaCl: Sodium chloride
SA: Salicylic acid
VIGS: Virus-induced gene silencing
VLCFA: Very-long-chain fatty acid
