# Supplementary material for: The cotton GhWIN2 gene activates the cuticle biosynthesis pathway and influences the salicylic and jasmonic acid biosynthesis pathways
Source: BMC Plant Biol. 2019 Aug 28;19:379. doi: 10.1186/s12870-019-1888-6 (PMC6712776; doi:10.1186/s12870-019-1888-6)
Supplement: Supplementary file 1 — Supplementary information. Figure S1 Amino acid sequence alignment of cotton WIN orthologs. Figure S2 Schematic representation of the plant expression vector pCAMBIA1300-GhWIN2. Figure S3 Relative expression of GhWIN2 in transgenic Arabidopsis plants. Figure S4 Expression of GhWIN2 in control and silenced cottons. Figure S5 VIGS-mediated silencing of GhCLA1 in cotton. Figure S6 Relative expression of GhPYL1 and GhNCED1 in cotton plants 14 days after agroinfiltration. Figure S7 Relative biomass of V. dahliae in infected cotton plants. Figure S8 VIGS-mediated silencing of GhICS2 in cotton. Table S1 Primers used in this study. (PDF 1037 kb) [file 12870_2019_1888_MOESM1_ESM.pdf]

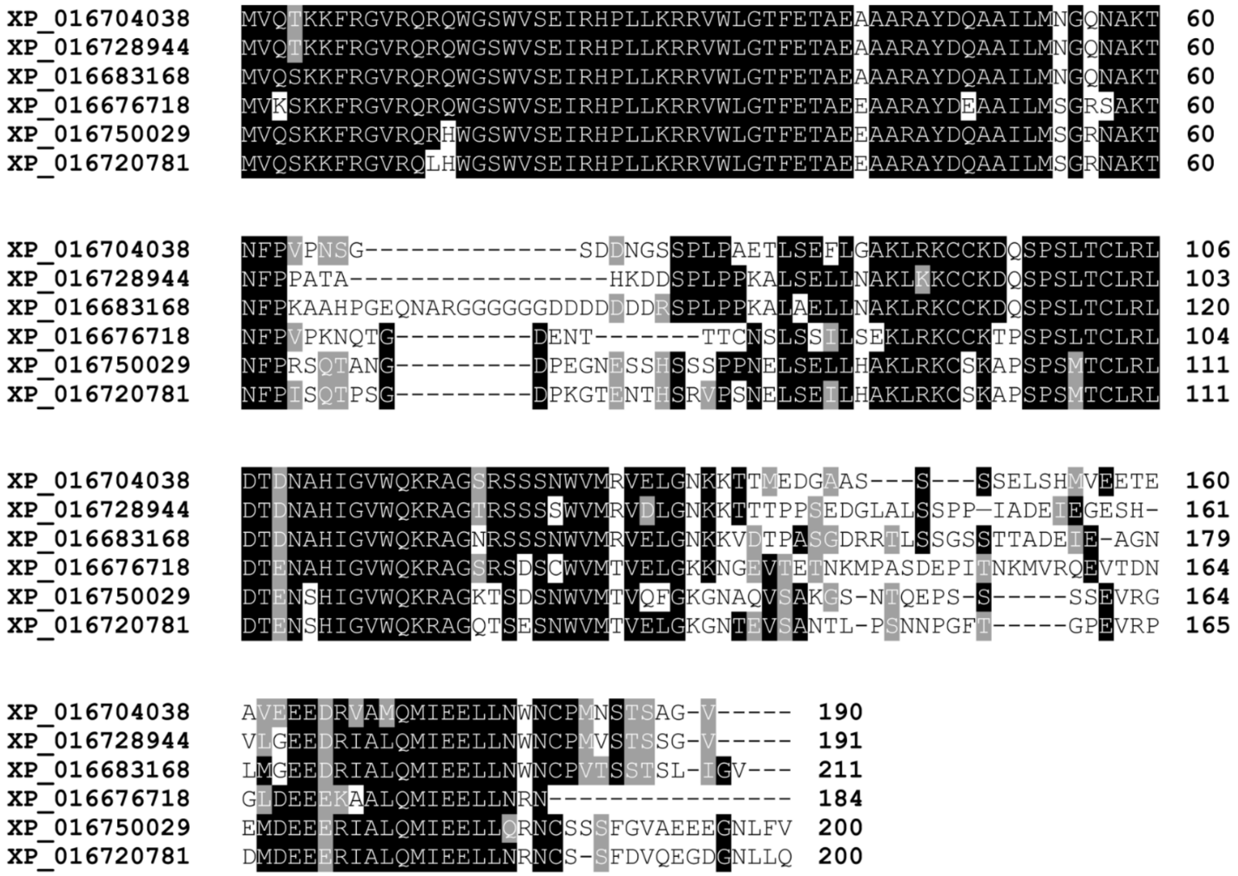

Fig. S1 Amino acid sequence alignment of cotton WIN orthologs. Black shading indicates 100% conservation, and gray shading denotes similar residues. Sequence alignment was performed with Clustal *W*.

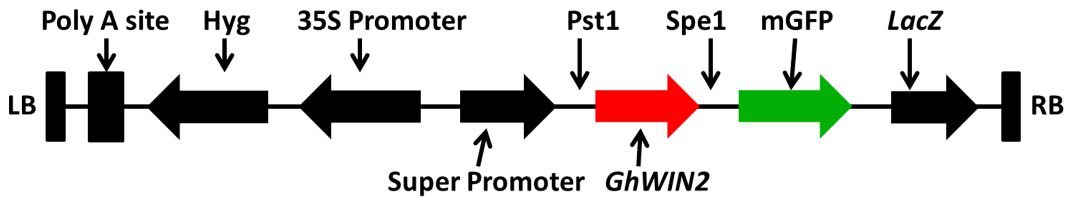

Fig. S2. Schematic representation of the plant expression vector pCAMBIA1300-*GhWIN2*.

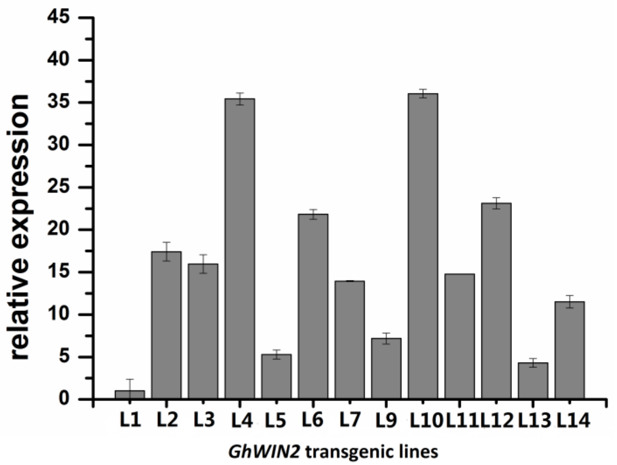

Fig. S3. Relative expression of *GhWIN2* in transgenic *Arabidopsis* plants. Data are presented as means ± SD from three independent biological replicates.

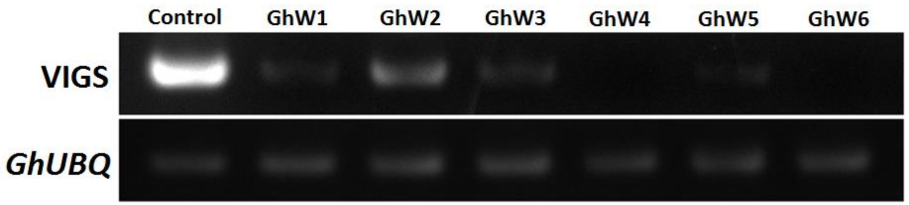

Fig. S4 Expression of *GhWIN2* in the control and silenced cottons were detected by semi-quantitative RT-PCR. *GhUBQ7* was used as an endogenous reference. The expression of *GhWIN2* in the RNAi lines (GhW1, GhW2, GhW3, GhW4, GhW5, and GhW6) was significantly lower than that in the control lines.

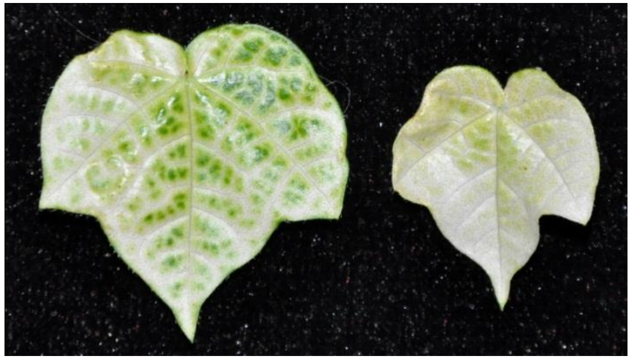

Fig. S5 VIGS-mediated silence of *GhCLA1* in cotton.

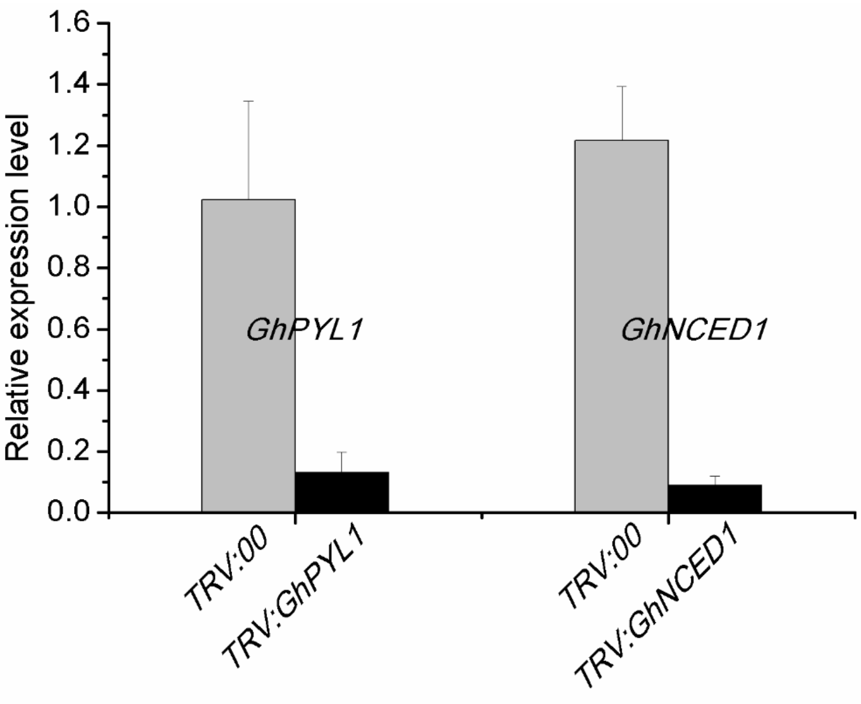

Fig. S6 Relative expression of *GhPYL1* and *GhNCED1* in cotton plants 14 days post agroinfiltration. Values are shown as means ± SD from at least three independent biological replicates.

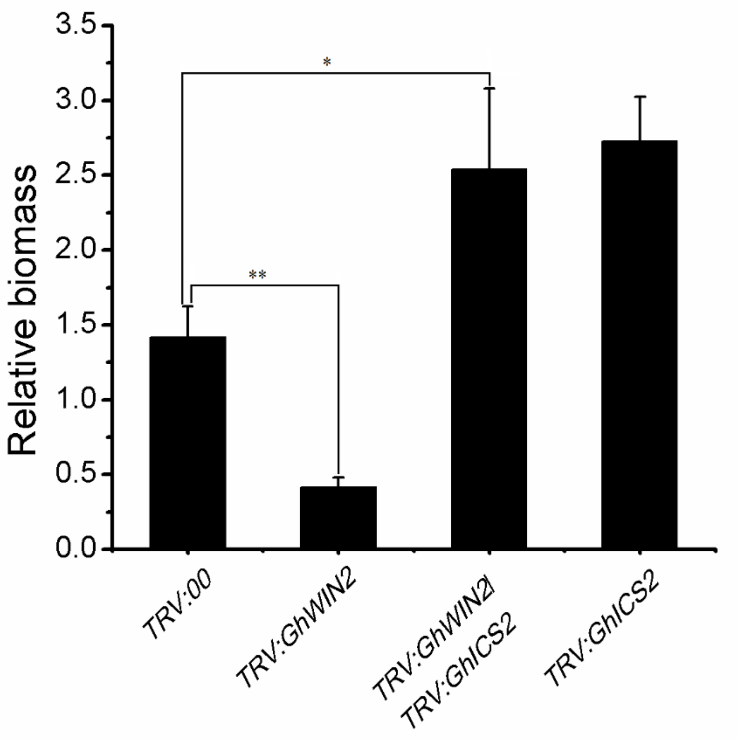

Fig. S7 Relative biomass of *V. dahliae* in infected cotton plants. Values are shown as means ± SD from at least three independent biological replicates.

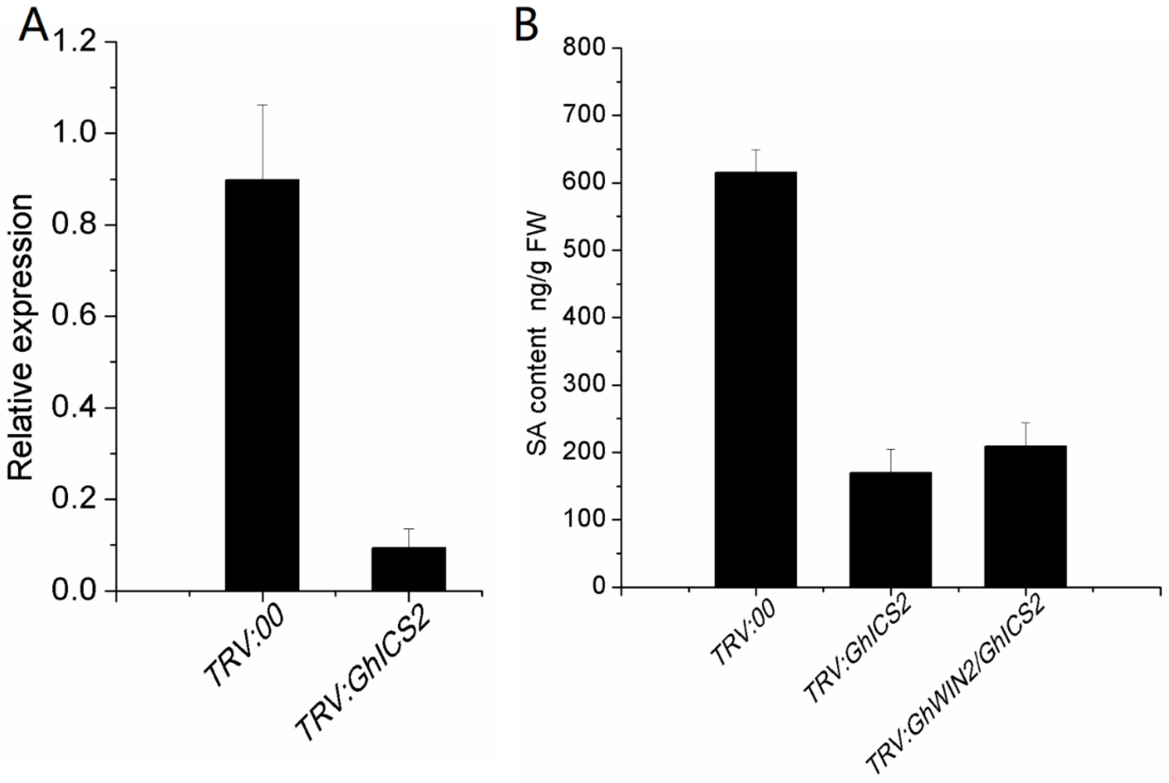

Fig. S8 VIGS-mediated silence of *GhICS2* in cotton. (A) Relative expression of *GhICS2* in *TRV:00* and *TRV:GhICS2* cotton plants 14 days post agroinfiltration. Values are shown as means ± SD from at least three independent biological replicates. (B) Content of SA in stem.

Table S1. Primers in this study

RT-qPCR (in cotton)	
GhWIN2-F	5'-CCAAGTGGAGACGCAAAAGGC-3'
GhWIN2-R	5'-TGGAAGGCAGCGTATTCGCTG-3'
GhUBQ7-F	5'-AGGCATTCCACCTGACCAAC-3'
GhUBQ7-R	5'-CAGCGAGCTTGACCTTCTTC-3'
GhCER1-F	5'-AGAGTGATGAGCGTATGGCG-3'
GhCER1-R	5'-GAGTTGGGACCTTCAGAGGC-3'
GhCYP86A4-F	5'- ATGCGGCTTGACTTTCGGTA-3'
GhCYP86A4-R	5'- GACTTCCATCCCAAGCCGAA-3'
GhLACS2-F	5'- GGATTTGCCAGACGGAATGC-3'
GhLACS2-R	5'- GAGAGCGGCATCATAAGCCT-3'
GhCYP86A7-F	5'- GATCCTATCAGCCGTGGTGG-3'
GhCYP86A7-R	5'- TGACAGTCACGAGACCTTGC-3'
GhGPAT6-F	5'-GGGTTTTGGTAGGGAAGTTTA-3'
GhGPAT6-R	5'-GGTTAGCAAGGCATTGAGTG-3'
GhCER3-F	5'-GACTGCACCTACGGTGATCT-3'
GhCER3-R	5'-CCTGCATGGCATGCATGCAC-3'
GhGPDHc1-F	5'-GCCTGAAGAATGTCTATGCC-3'
GhGPDHc1-R	5'-CCGCTGATGCTATCACCCAT-3'
GhCER6-F	5'-CCACAAGGGAGCTTCTCAAGG-3'
GhCER6-R	5'-GCTACTTGCCGATGTGTTGCC-3'
GhAOC5-F	5'-GGGATAATCAGATCAACTTC-3'
GhAOC5-R	5'- GCTAAAAGGGACAAGATCAC-3'
GhAOS-F	5'- TTAACTTCTTGTGCCGGGCT-3'
GhAOS-R	5'- TTTAACCAAAGCCGGGGGAA-3'
GhDCD1-F	5'-TTTGAGAGGCACCGCTACTG-3'

GhDCD1-R	5’-TTTCCACCATGGGTTCCGAG-3’
GhLOX1-F	5’- AGAACTCCGAGAGGAGGGTC-3’
GhLOX1-R	5’- CGGGAGGTAGCCTGCATAAG-3’
GhOPR3-F	5’-GCTTACGGGCAAACCGAATC-3’
GhOPR3-R	5’-AGGCGGCCGTAAGATACAAG-3’
qGhFATA-F	5’-TGCATCTCATATGGGTGA-3’
qGhFATA-R	5’-TTCTCGTGGACAGAAGAC-3’
qGhFATB-F	5′-TGCTGCTATCACAACCAT-3′
qGhFATB-R	5′-CCTGAACAATCTTCCCTAT-3′
qGhSAD3-F	5’-CCAGTGTTCCATTCAGTT-3’
qGhSAD3-R	5’-CCAGTTGTTTCTTTCTTGTAT-3’
qGhSAD7-F	5’-GCTGGATATTTGATAGAGAAG-3’
qGhSAD7-R	5’-CAGGAAATTGAAATCACCAT-3’
GhKASII-F	5’-TCGAGACTTTTGACTGCGCT-3’
GhKASII-R	5’-TGCAAAGCTTTCTTTCCGGC-3’
ITS1-F	5’-AAAGTTTTAATGGTTCGCTAAGA-3’
ST-VE1-R	5’-CTTGGTCATTTAGAGGAAGTAA-3’
**RT-qPCR (in *Arabidopsis*)**	
EF-1-α-F	5'-CCTGGATTGCCACACC-3'
EF-1-α-R	5'-AGTCTGCCTCATGTCC-3'
AtKCS1-F	5’-TCCTCTGCAACTGCATCTTCCG-3’
AtKCS1-R	5’-CGACAGACATGAGCTCTCTAGC-3’
AtGPAT6-F	5’-ACTTTCCTCTGGCTTCCCGTCG-3’
AtGPAT6-R	5’-ATCGAGAACGGTGCGGTGGTTG-3’
AtCER6-F	5’-GTGAGGAGACTTGTCTCCCTCC-3’
AtCER6-R	5’-TAGCTGAGAGCGATGGTGTGGG-3’
AtCER3-F	5’-TGAGGCTGCGATTCTTAGAGCTG-3’
AtCER3-R	5’-CCACACGAACTCTAAGGTCAGG-3’

AtLACS2-F	5’-GATCGATCGTGTGACACGAGCG-3’
AtLACS2-R	5’-CCACGCCAGTATCCAACAGAGG-3’
AtCYP86A4-F	5’-ACGGCTCAAGACAATGCAGA-3’
AtCYP86A4-R	5’-TAAACCGTTGAAGCGAGGCT-3’
AtGPDHc1-F	5’- TCATCAAGCAACTGGTGTGCCG -3’
AtGPDHc1-R	5’- TACCAGCTCCAATGGCGTAGAC -3’
AtCER6-F	5’-GTGAGGAGACTTGTCTCCCTCC-3’
AtCER6-R	5’-TAGCTGAGAGCGATGGTGTGGG-3’
AtCER1-F	5’-ATACCACTCGCTGCACCACACG-3’
AtCER1-R	5’-TGGATTCTGGCGTCGTCAGGTG-3’
AtCER2-F	5’-GGTCCTGATCTTACCTTCTCGC-3’
AtCER2-R	5’-ATGACCCGATACCAGCTGTCCA-3’
**VIGS**	
GhWIN2-F	5’-CgACgACAAgACCgTgACCATgGTTAGGCAGCGACACTGG-3’
GhWIN2-R	5’-gAggAgAAgAgCCgTCATTAGAGAAGGTGCCTTGCTGC-3’
GhCLA1-F	5`-CGACGACAAGACCGTGACCATGCACAACATCGATGATTTAG-3`
GhCLA1-R	5`-GAGGAGAAGAGCCGTCATTAGCATGAATGATGAGTAGATTGCAC-3
GhPYL10-F	5’-CgACgACAAgACCgTgACCATgCACCGTCTGGTCCGTTGTC-3’
GhPYL10-R	5’-gAggAgAAgAgCCgTCATTAGTGTCGACGAACACACAGG-3’
GhNCED1-F	5’-CgACgACAAgACCgTgACCATgTGAGCTTCAACATCCACTTCC-3’
GhNCED1-R	5’-gAggAgAAgAgCCgTCATTAACTAAGCCAGCATTAGCTACC-3’
GhICS2MO-F	5’-CgACgACAAgACCgTgACCATgGATCGAGGAGCAAATCGAAGC-3’
GhICS2MO-R	5’-gAggAgAAgAgCCgTCATTAAGGGCATTGTCCCATGCAATC-3’
**Transgenic assay**	
WIN-1300-F	5’-AAACTGCAGATGGTGCAATCAAAGAAG-3’
WIN-1300-R	5’-TAGACTAGTCTGCAACAAGTTACCATC-3’
**Transient expression assay**	

WIN-pROK2-F	5'-CCC TCTAGA ATGGTGCAATCAAAGAAGTT-3'
WIN-pROK2-R	5'-CCC GGATCC CTGCAACAAGTTACCATCTC-3'
WIN^{V-A}-F	5'-GGCTCCTGGGTGTCTGAAATTCGCCATCC-3'
WIN^{V-A}-R	5'- GAATTTCAGACACCCAGGAGCCCCAGTGTC-3'
GhCYP86A4$_{pro}$-F	5'-AATTATATAACGTGGATAC-3'
GhCYP86A4$_{pro}$-R	5'-ACTATTACAACTATAACAA-3'
